# Function of the Shaw Potassium Channel within the *Drosophila* Circadian Clock

**DOI:** 10.1371/journal.pone.0002274

**Published:** 2008-05-28

**Authors:** James J. Hodge, Ralf Stanewsky

**Affiliations:** 1 Department of Physiology and Pharmacology, School of Medical Sciences, Bristol University, Bristol, United Kingdom; 2 School of Biological and Chemical Science, Queen Mary University of London, London, United Kingdom; The Rockefeller University, United States of America

## Abstract

**Background:**

In addition to the molecular feedback loops, electrical activity has been shown to be important for the function of networks of clock neurons in generating rhythmic behavior. Most studies have used over-expression of foreign channels or pharmacological manipulations that alter membrane excitability. In order to determine the cellular mechanisms that regulate resting membrane potential (RMP) in the native clock of *Drosophila* we modulated the function of Shaw, a widely expressed neuronal potassium (K^+^) channel known to regulate RMP in *Drosophila* central neurons.

**Methodology/Principal Findings:**

We show that Shaw is endogenously expressed in clock neurons. Differential use of clock gene promoters was employed to express a range of transgenes that either increase or decrease Shaw function in different clusters of clock neurons. Under LD conditions, increasing Shaw levels in all clock neurons (LNv, LNd, DN_1_, DN_2_ and DN_3_), or in subsets of clock neurons (LNd and DNs or DNs alone) increases locomotor activity at night. In free-running conditions these manipulations result in arrhythmic locomotor activity without disruption of the molecular clock. Reducing Shaw in the DN alone caused a dramatic lengthening of the behavioral period. Changing Shaw levels in all clock neurons also disrupts the rhythmic accumulation and levels of Pigment Dispersing Factor (PDF) in the dorsal projections of LNv neurons. However, changing Shaw levels solely in LNv neurons had little effect on locomotor activity or rhythmic accumulation of PDF.

**Conclusions/Significance:**

Based on our results it is likely that Shaw modulates pacemaker and output neuronal electrical activity that controls circadian locomotor behavior by affecting rhythmic release of PDF. The results support an important role of the DN clock neurons in Shaw-mediated control of circadian behavior. In conclusion, we have demonstrated a central role of Shaw for coordinated and rhythmic output from clock neurons.

## Introduction

A 24 hour (circadian) cycle of rest and activity exists in almost all animals, persisting even in complete darkness. In flies, this behavior is dependent on the rhythmic expression of oscillating genes under control of a molecular clock that consists of interlocked molecular feedback loops of transcription including the *period* (*per*) and *timeless* (*tim*) genes. These feedback loops, and the behavioral rhythms controlled by them, can be reset cell-autonomously by light-induced TIM degradation via the blue-light photoreceptor protein Cryptochrome (CRY) and through light inputs to the pacemaker neurons from the eyes. This central molecular clock mechanism operates in a set of pacemaker neurons located in the fly brain [1].

Control of electrical activity has been postulated to be important for the function of the clock [2]–[3]. Studies in the mammalian suprachiasmatic nucleus (SCN) show that cell-autonomous, circadian-oscillatory expression of clock genes drives the circadian rhythms in neuronal firing rate and resting membrane potential (RMP) [4], [5]. The mechanism of these oscillations in mammals and *Drosophila* may involve ion channels under direct transcriptional control of the clock gene products or post-translational modulation of ion channels by clock controlled proteins [6]–[8]. Voltage-gated K^+^ channels are key regulators of the intrinsic excitability in all neurons, and therefore they are crucial for output rhythms of clock neurons under free-running conditions in the *Bulla* eye [9]–[10] and in the SCN [11]–[12], as well as for molecular clock-gene oscillations inside the nucleus and cytoplasm of SCN neurons in mammals [13]. The role of endogenous K^+^ channels has not been extensively explored in *Drosophila* central clock neurons.

Current models of the mammalian SCN predict RMP to be rhythmically regulated by the clock [3]. In *Drosophila* artificial expression of truncated dORK channels has also been shown to hyperpolarize RMP and to decrease the firing rate of clock neurons [14]. The same treatment results in behavioral arrhythmicity under constant conditions and a loss of clock protein cycling in pacemaker cells. Therefore these studies used over-expression of foreign channels to modulate electrical activity and constitutive hyperpolarization or depolarization of clock cells was found to be disruptive to clock function in constant conditions [15]–[17].

These studies did not, however, shed any light on the actual cellular mechanisms that regulate RMP in the native clock. So far it is not known which endogenously expressed ion channels and membrane proteins influence electrical membrane properties of *Drosophila* clock neurons or if they are regulated by the circadian clock. A number of *Drosophila* ion channels have been implicated in clock function, these include Narrow Abdomen (NA), a Na^+^/Ca^++^ channel [18] and Slowpoke (SLO), a Ca^++^-sensitive K^+^ channel [19] whose mammalian homolog (BK) is also important for circadian regulation [20].

These studies suggest that electrical membrane properties maybe similarly important in the fly clock and mammalian SCN. Moreover, mammalian Shaw homologues, Kv3 channels, are widely distributed within the SCN and the magnitude of their current varies between the day and the night, even under free-running conditions. Blocking Kv3.1b and Kv3.2 currents prevents the daily rhythm in firing of SCN neurons [21]. In *Drosophila*, Shaw is a member of the Shaker family of voltage-gated K^+^ channels and encodes a slowly activating and non-inactivating K^+^ current. These channels can be open at normal cell RMP and cause hyperpolarization by allowing K^+^ efflux. In *Drosophila*, the Shaw K^+^ channel is widely expressed in the nervous system and helps regulate RMP in *Drosophila* central neuron [22]–[25]. Shaw is therefore an attractive K^+^ channel to endogenously regulate RMP in clock neurons. In this study we reveal a circadian function of Shaw K^+^ channels in *Drosophila* and demonstrate that they are required for rhythmic output from clock neurons.

## Materials and Methods

### Fly strains

Flies were grown at similar density in bottles on standard medium at 25°C in 12 hr∶12 hr LD cycles. The following strains were used: *CantonS*, *Pdf-GAL4* [26]–[27], *tim-GAL4* insert 27 and 67 [27], *cry-GAL4* insert 13 [18], [28]–[29], *8.0-luc:9D* [30], *UAS-Shaw-FLAG* insert 12B and *UAS-ShawTRuncated-FLAG* insert 332 (truncated at residue 369) [25]. Data were confirmed with a second insert of each *Shaw* transgene. The *Pdf-GAL80* and *cry-GAL80* [18], [28]–[29] lines were a kind gift of Dr. Patrick Emery (U. Mass., Worcester).

### Transgenic lines

The *UAS-Shaw RNAi* construct contains a 720 bp fragment of the 3′ end of Shaw starting at nucleotide 881 in Exon 8 through to the end of the gene including approximately 110 bp of 3′ untranslated sequence. This fragment was cloned into the multiple cloning site of the SympUAST transformation vector [31] that was then introduced into the *Drosophila* germline by *P*-element mediated transformation. Stable lines were established and a line containing the insert homozygous on both chromosome *2* and *3* was used.

### Behavior

Locomotor activity of adult males placed in an infrared beam-cross counting apparatus was monitored automatically and analyzed as in Wülbeck et al. [32] using MATLAB analysis software [33]. Flies were raised in 12 hr∶12 hr LD cycles at 25°C and then assayed for locomotor activity for the next 7 days in LD. This was followed by 9 to 16 days in constant darkness (DD).

### Immunohistochemistry, imaging and quantification

Whole-mounted adult brains were processed and stained with a rabbit anti-Shaw C terminal (amino acids 413–498) antibody (pre-absorbed and used at 1∶1000), mouse anti-FLAG (affinity purified M2 F1804 used at 1∶1000, Sigma-Aldrich, St. Louis MO), rabbit anti-crab PDH (affinity purified used at 1∶1500, [34]) and Alexa488, Alexa594 (1∶180; Molecular Probes, OR), FITC and Cy5 secondary antibodies (1∶180; Jackson, West Grove PA) using standard immunohistochemical techniques [25], [35].

All preparations were processed in parallel and images acquired with identical settings using the 40× or 50× (zoomed 1–4×) objectives of a Leica TCS SP2 confocal microscope. Care was taken to keep all intensity readings within the linear range below saturation and all double immunofluorescent images were scanned sequentially. Quantification was performed on 1 µm sections with pixel intensity readings taken in a given region of interest for PDF-Alexa488 (10 LNv axon terminal boutons per hemisphere) with a surrounding background measurement subtracted using the Leica TCS SP2 quantification software. Quantification was performed blind to genotype and experimental condition. Statistical analysis was performed in Excel (Microsoft) and JMP (SAS). Significance levels in figures were determined by one-way ANOVA unless otherwise specified.

### Analysis of Bioluminescence Rhythms

Luciferase expression of individual flies carrying the *8.0-luc:9D* transgene was measured, analyzed, and plotted as described in Veleri et al. [30]. Prior to each experiment, flies were entrained for at least 2 days to a 12 hr∶12 hr LD cycle at 25°C and kept in the same regime for the first 2 days of the experiment. Subsequently flies were subjected to constant conditions (DD) for 3–5 days.

### Fly head extracts

2–3 day old flies were entrained to 12 hr∶12 hr LD cycles for 2–3 days and frozen in liquid nitrogen. After decapitating by vortexing, equal numbers of frozen heads from each genotype were homogenized and solubilized in ice-cold RIPA buffer for 30 min. Homogenates were spun at 500× *g* and then 14,000× g for 15 min at 4°C to remove debris [35]. Protein was separated by SDS-PAGE, and analyzed by immunoblot using rabbit anti-C terminal Shaw antibody (1∶1000; [25]) and rabbit anti-PER (1∶10,000; [32]).

## Results

### Shaw is expressed in clock neurons

**Figure 1:**
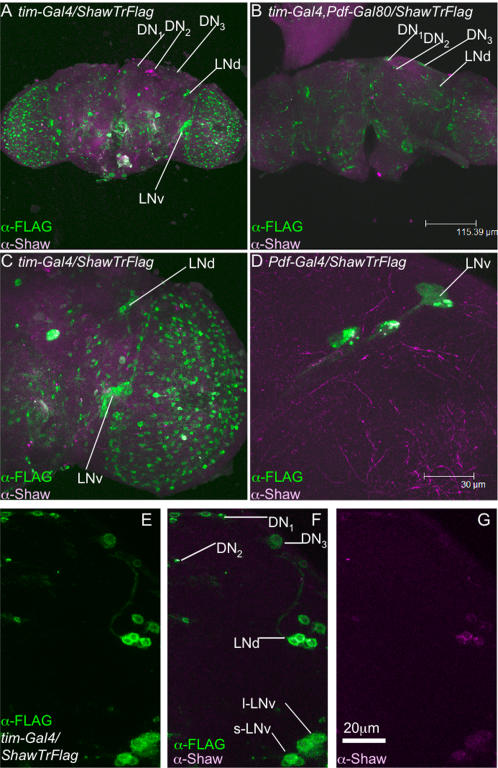
Shaw is widely expressed in the adult brain including in a subset of clock neurons. The clock neurons are divided into lateral neurons (LN) and dorsal neurons (DN). The LN's consist of 6 dorsally located neurons (LNd) and two cell clusters of ventrally located neurons [4–6 large LNv (l-LNv) and 5 small (s-LNv)]. DN cells are divided into ∼15 DN_1_, 2 DN_2_ and ∼40 DN_3_, which also differ in size and position. The DN_1_ and DN_3_ project to the s-LNv and l-LNv. The s-LNv projections terminate in the dorsal part of the brain while the l-LNv projections terminate on the surface of the medulla and close to the LNv of the contralateral brain hemisphere. The LNv pacemaker cells (except for one) produce a neuropeptide, Pigment Dispersing Factor (PDF) that is likely to function as a circadian output signal [36]. (A), (C) and (E–G) *tim-GAL4; UAS-Shaw-Truncated-FLAG* stained with anti-FLAG and green secondary antibody reveals the mostly cytoplasmic localized truncated channel [25] in the *tim* complement of clock neurons [27]. An antibody to the C terminus of Shaw (downstream of the truncation) detects endogenous Shaw [25] co-localized (in white) in a subset of each cluster of clock neurons, while magenta shows Shaw in and around *tim* clock neurons. (B) *Pdf-GAL80* was used to subtract expression of the Shaw transgene in LNv clock neurons from the remainder of the *tim-GAL4* pattern, leaving expression in the DN and LNd neurons intact [18], [28]–[29]. (D) A *Pdf-GAL4; UAS-Shaw-Truncated-FLAG* brain double-labeled with anti-FLAG and anti-Shaw. The dominant negative Shaw subunits seem to aggregate endogenous Shaw subunits in clusters in the PDF neuronal cell bodies, thereby making it possible to visualize endogenous Shaw.

To determine if Shaw is expressed in the cells that constitute the circadian clock, we used whole-mount immunohistochemistry and confocal imaging (Figure 1). Clock neurons can be visualized and genetically accessed by using *GAL4* drivers [37] that are controlled by clock-cell specific promoters such as those from the *timeless* (Figure 1A, C and E–G) and *Pdf* (Figure 1D) genes [26]–[27]. *tim-GAL4* is expressed strongly in all clock neurons including the DN_1_, DN_2_, and DN_3_ groups and in the well characterized dorsal and ventral lateral neurons (LNd and LNv). *Pdf-GAL4* is expressed only in the small and large ventral lateral neurons (s- and l-LNv). Using GAL80 to suppress GAL4 activity [38], it is possible to subdivide these expression patterns further. In *tim-GAL4*; *Pdf-GAL80* animals, only LNd and Dorsal Neurons should express UAS transgenes [18], [28]–[29]. However, after careful inspection of our data, we cannot rule out that even in the presence of *Pdf-GAL80* weak reporter gene expression (of the Flag-epitope tagged, truncated version of Shaw, ShawTR: [25]) remains in at least some of the l-LNv (Figure 1 compare A and B). The large size and position of the weakly staining cell bodies suggests that these are not the small LNv, which are generally considered to be more circadianly relevant as they maintain molecular clock oscillation in DD, as compared to the large LNv [26], [29], [39]–[40].

To visualize endogenous Shaw expression, whole brains of the *tim-GAL4/UAS-ShawTR* flies were double-labeled with an antibody directed against the C-terminus of Shaw (absent from the truncated transgene, in magenta, Figure 1) and anti-Flag antibodies. Endogenous Shaw was found to be in and close to a number of clock neurons (co-expression indicated by the white signal, Figure 1D–F) including the s- and l-LNv, LNd and at a low level in at least some of the DN (Figure 1B and G). In *Pdf-GAL4* animals, endogenous Shaw subunits are sequestered into cytoplasmic clusters (in white), likely due to the ability of the endogenous and truncated channels to multimerize and the fact that the C-termini of Shaw channel complexes are required for correct localization [25], [41]. ShawTR has been demonstrated to generate Shaw dominant negative phenotypes using both electrophysiological and behavioral assays presumably by assembling with endogenous Shaw subunits resulting in a non-functioning tetrameric channel [25]. These data suggest that ShawTR effectively traps endogenous Shaw in cytoplasmic inclusions and demonstrate that endogenous Shaw is expressed in a subset of clock neurons. Expression of ShawTR does not appear to change the number, or disturb the viability or morphology of these neurons (Figure 1 and data not shown).

To explore the role of Shaw in the clock we employed the *GAL4/UAS* system to manipulate channel function and level in different subsets of clock neurons. Three approaches were used: i) targeted over-expression of Shaw using a full-length transgene; ii) targeted expression of a dominant-negative C-terminal truncated form of Shaw (ShawTR, see above) ([25]; Figure 1); and iii) reduction of Shaw levels by expressing *ShawRNAi*. In animals expressing the latter transgene, Shaw levels are significantly decreased both when analyzed by immunoblotting and when endogenous Shaw levels are quantitatively assessed immunohistochemically in the intact brain (L.C. Griffith, personnel communication).

### Over-expression of Shaw in the clock alters locomotor activity in LD

In order to determine if Shaw has a specific role in regulating rhythmic behavior, we measured locomotor activity in 12 hr∶12 hr light∶dark (LD) cycles. To increase non-inactivating K^+^ current in clock neurons, full-length Shaw was over-expressed in the *tim-GAL4* pattern (Figure 1). This would be expected to hyperpolarize the RMP, thereby reducing spontaneous firing of action potentials in the clock neurons in which it is expressed (14,25). To decrease the Shaw mediated non-inactivating K^+^ current, *ShawTR* and *ShawRNAi* were expressed. The functional effect of these manipulations is a depolarization of the RMP, expected to result in an increase in spontaneous firing of action potentials in the neurons in which it is expressed (Figure 1; [25] and L.C. Griffith, personnel communication).

**Figure 2:**
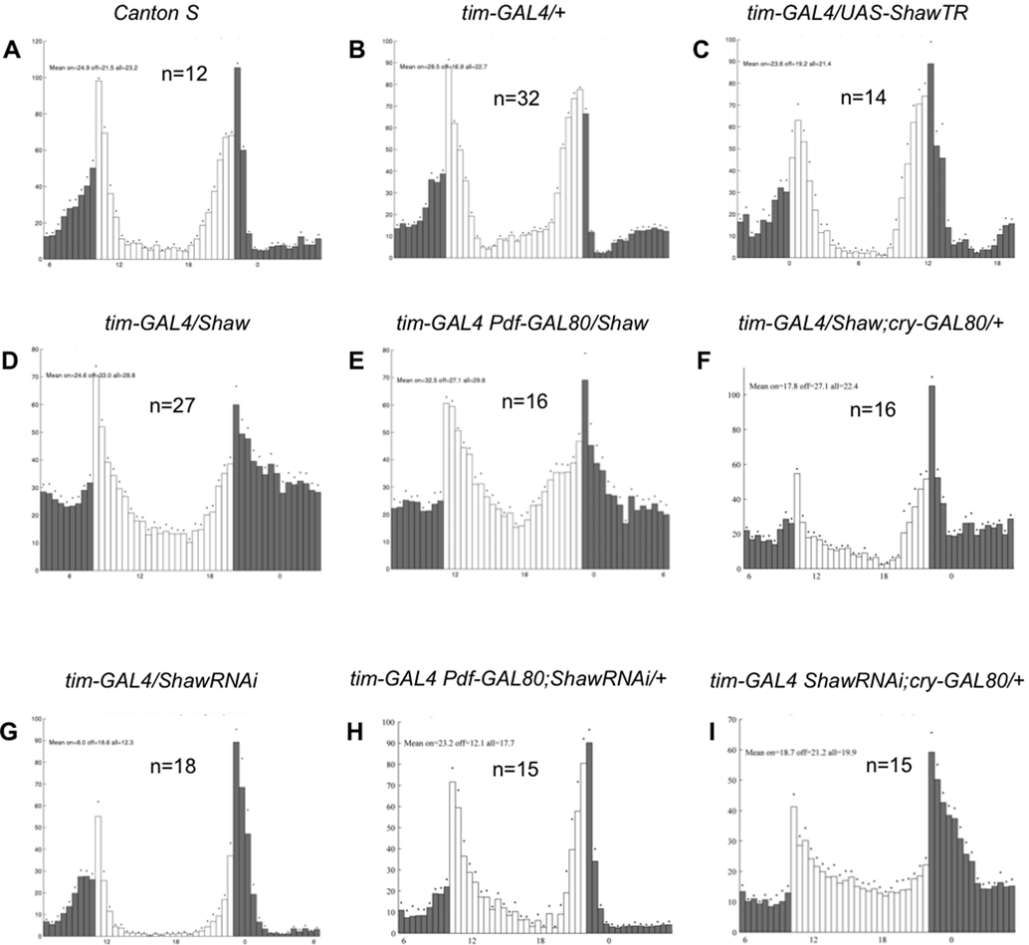
Over-expression of Shaw increases activity during the night. Histograms show daily averages of locomotor activity in the LD portion of the experiment (7 days). Open and black bars indicate activity levels during 30 min intervals when the lights were on and off, respectively. All genotypes exhibit bimodal behavior, showing the characteristic anticipation of the lights-on transition in the morning, and the lights-off transition in the evening. Only the LD behavioral pattern of the *tim-GAL4 ShawRNAi/+; cry-GAL80/+* is altered, perhaps due to a more fundamental disturbance of clock function (see text for details). SEM's are indicated by dots above each histogram bar.

Increasing Shaw in all the clock cells (*tim-GAL4/UAS-Shaw*; Figure 2D) caused flies to have more locomotor activity compared to controls (Figure 2A–B). Determining the overall average activity for the day and night portion of the experiment respectively, revealed that activity was significantly increased at night only (Table 1). Adding *Pdf-GAL80* to *tim-GAL4/UAS-Shaw* to remove *Shaw* over-expression in the LNv has no effect on the hyperactive *tim-GAL4/UAS-Shaw* phenotype (Figure 2, compare E to D; Table 1). Surprisingly, even after restricting *Shaw* over-expression to the Dorsal Neurons (*tim-GAL4/UAS-Shaw; cry-GAL80*) increased night activity persisted (Figure 2F, Table 1). Together this indicates that clock-cell specific *Shaw* expression in *Pdf ^−^* and *Cry^−^* cells increases activity in LD. Similarly, *cry-GAL4* (but not *Pdf-GAL4*) driven *Shaw* over-expression resulted in increased activity at night, suggesting that the *cry* expressing Dorsal Neurons also contribute to this phenotype (Figure S1C–D; Table 1). However, when *Shaw* expression in the *cry-GAL4* flies is excluded from the *Pdf* cells (*cry-GAL4/UAS-Shaw; Pdf-GAL80*), no activity increase was observed, indicating that the *Pdf ^+^* cells also contribute to this phenotype (Figure S1G; Table 1). We have no explanation why *Shaw* expression in the Dorsal Neurons is not sufficient to increase night activity in this latter genotype, except that the *cry^+^* DNs contribute to this phenotype to a lesser extent compared to the *cry^−^* ones (and require *Shaw* expression in other clock neurons as well as for example in the LNv in the *cry-GAL4* flies). In summary, our results indicate that the DNs are sensitive to *Shaw* levels, and to some extent the LNv too. These findings are in good agreement with the endogenous Shaw expression detected in these cells (Figure 1).

**Table 1:** Average activity levels during 12 hr: 12 hr light dark cycles.

Genotype	n	day	night	total
*Canton-S*	12	24.9	21.5	23.2
*tim-GAL4/UAS-Shaw*	27	24.6	33.0	28.8
*cry-GAL4/UAS-Shaw*	12	36.2	47.6	41.9
*Pdf-GAL4/UAS-Shaw*	28	27.9	22.2	25.1
*tim-GAL4, Pdf-GAL80/UAS-Shaw*	16	32.5	27.1	29.8
*tim-GAL4/UAS-Shaw; cry-GAL80/+*	16	17.8	27.1	22.4
*cry-GAL4, Pdf-GAL80/UAS-Shaw*	23	25.0	21.5	23.3
*tim-GAL4/UAS-ShawTR*	14	23.6	19.2	21.4
*cry-GAL4/UAS-ShawTR*	13	21.5	22.0	21.8
*Pdf-GAL4/UAS-ShawTR*	15	28.9	24.4	26.7
*cry-GAL4, Pdf-GAL80/UAS-ShawTR*	15	24.0	22.8	23.4
*tim-GAL4 Pdf-GAL80/UAS-ShawTR*	15	27.5	20.2	23.9
*tim-GAL4/UAS-ShawTR; cry-GAL80/+*	4	27.1	38.7	32.9
*tim-GAL4/UAS-ShawRNAi*	18	8	16.6	12.3
*tim-GAL4 Pdf-GAL80/UAS-ShawRNAi*	15	23.2	12.1	17.7
*tim-GAL4 ShawRNAi/+; cry-GAL80/+*	16	18.7	21.2	19.9
*tim-GAL4 ShawRNAi/+; cry-GAL80/cry-GAL80*	7	11.6	16.0	13.8
*tim-GAL4/+*	32	28.5	16.9	22.7
*cry-GAL4/+*	16	31.7	26.7	29.2
*Pdf-GAL4/+*	28	23.6	23.8	23.7
*UAS-Shaw/+*	16	26.1	15.6	20.8
*UAS-ShawTR/+*	15	29.8	15.0	22.4
*UAS-ShawRNAi/+*	14	16.6	14.5	15.5
*UAS-Shaw/+; cry-GAL80/+*	14	22.5	9.7	16.1

Numbers in the ‘day’ and ‘night’ columns indicate the average activity (beam crossings/30 min) of each fly of a given genotype during that part of the day. It is calculated based on the activity displayed during each 30 min ‘bin’ during the whole experiment (usually 7 days) that was also used to generate the daily average histogram plots shown in Figure 2 and Figure S1. So, for example each entry in the ‘day’ column is an average of a given fly's activity during 30 min in the light portion of 7 days, which is again averaged among all the flies tested for this genotype (n). The SEM for each 30 min interval of the light and dark portion for the 7-day average is indicated in Figure 2 and Figure S1.

In contrast to *Shaw* over-expression, reducing *Shaw* function by expressing the dominant-negative *ShawTR*, or reducing Shaw levels by expressing *ShawRNAi* in all clock neurons had no clear effect on locomotor rhythms in LD (Figure 2C, G; Table 1). Although it appeared initially that *tim-GAL4/UAS-ShawRNAi* flies exhibited reduced activity levels during the day and night (Figure 2G), this behavior is likely attributable to the genetic background of the *UAS-ShawRNAi* line (Table 1). As expected from these results, no clear effects on LD behavior could be observed when *ShawRNAi* or *ShawTR* expression was restricted to the *Pdf ^−^* cells (Figure 2H, and Figure S1H; Table 1) or to the *Pdf ^+^* and *cry^+^* cells, respectively (Figure S1E–F; Table 1, and data not shown).

A striking and surprising alteration from the wild-type behavior pattern was observed when *ShawRNAi* expression was restricted to the *cry^−^* cells—a large fraction of the DNs. Silencing endogenous *Shaw* expression in the DN mediated by *ShawRNAi* resulted in a lack of behavioral anticipation of the ‘lights-on’ transition in the morning, and only a marginal anticipation of the ‘lights-off’ transition in the evening (Figure 2I). But instead of a specific defect in LD behavior, this abnormal behavioral pattern is most likely caused by a more central clock or clock-output defect, resulting in a drastically increased free running period (see below).

### Changing levels of Shaw K^+^ channels in the clock results in arrhythmic locomotor behavior or pronounced period changes in free running conditions

**Figure 3:**
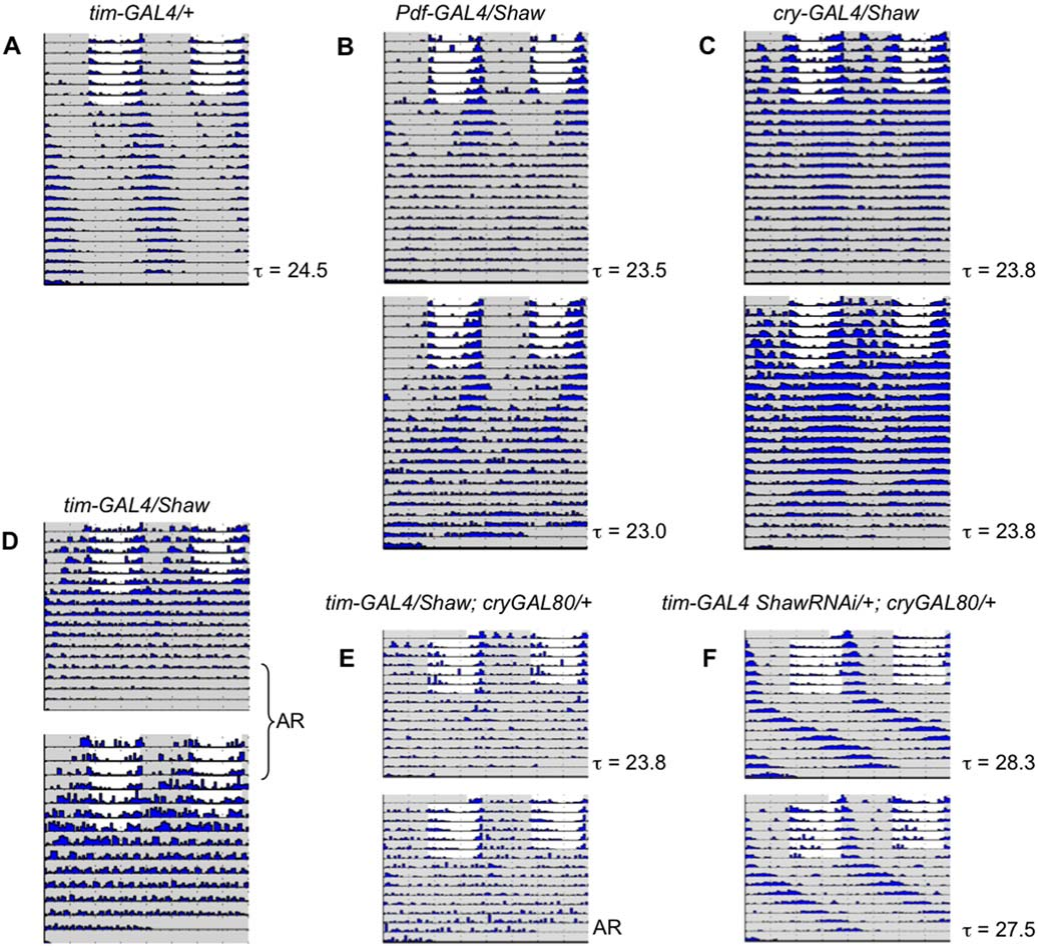
Effects of *Shaw* on free-running behavior. Locomotor activity of individual flies was measured for 7 days in LD followed by 9–16 days in DD and plotted as actograms. Shaded and white areas indicate when lights were off and on, respectively. The height of the black bars on each line of the actogram correlates with the activity level of the fly (measured in 30 min intervals). Free running period length (τ) for the DD part of the experiment is indicated below each actogram. To better visualize rhythmic behavior, each row of an actogram represents two consecutive days (double plot). Note that *tim-GAL4/UAS-Shaw* flies (D) are arrhythmic (AR; see also Table 2) compared to *Pdf-GAL4/UAS-Shaw* flies (B) and a control: *tim-GAL4/+* (A) (other control flies showed a similar behavior; see also Table 2). Restricting expression of Shaw to the majority of dorsal neurons (*tim-GAL4; cry-GAL80*) caused a significant proportion of the flies to be arrhythmic (E), while expression of Shaw-RNAi in these cells (F) caused a dramatic and significant lengthening of the free-running period.

To test the ability of flies to maintain their circadian rhythm in constant conditions, flies were monitored in constant darkness (DD) for 9–16 days following the LD portion of the experiment (Figure 3). Expression of *Shaw* using *tim-GAL4* results in arrhythmic (AR) behavior immediately after the transition to DD (Figure 3D; Table 2). Moreover, *tim-GAL4/UAS-Shaw* flies exhibited a reduced lifespan, with individuals never surviving until the end of an experiment (compare Figure 3D with Figure 3A–C). Flies that express Shaw less extensively in the clock network (*cry-GAL4/UAS-Shaw* or *Pdf-GAL4/UAS-Shaw*) maintain rhythmicity in DD albeit with reduced amplitude compared to controls (Figure 3B–C, and Table 2). It is also evident that over-expression of Shaw results in higher activity levels, similar as observed in LD (Figure 2D, Table 1).

**Table 2:** Shaw levels in clock neurons influence rhythmic behavior in constant darkness.

Genotype	n	% Rhythmic	τ (h)	Rhythm Strength (RS)
*w; Canton-S*	20	95	23.7±0.1	6.2±0.4
*tim-Gal4/UAS-Shaw*	39	13	24.4±0.4	2.5±0.2
*cry-Gal4/UAS-Shaw*	34	88	23.8±0.1	6.2±0.4
*Pdf-Gal4/UAS-Shaw*	27	89	23.7±0.1	5.5±0.4
*tim-Gal4, Pdf-Gal80/UAS-Shaw*	15	20	23.9±0.2	2.5±0.2
*tim-Gal4/UAS-Shaw; cry-Gal80/+*	15	67	24.0±0.1	3.8±0.4
*cry-Gal4, Pdf-Gal80/UAS-Shaw*	10	100	23.8±0.2	5.6±0.8
*tim-Gal4/UAS-ShawTR*	46	50	24.6±0.2	2.7±0.2
*cry-Gal4/UAS-ShawTR*	23	91	24.2±0.1	5.9±0.5
*Pdf-Gal4/UAS-ShawTR*	15	100	24.2±0.1	8.3±0.6
*cry-Gal4, Pdf-Gal80/UAS-ShawTR*	11	100	24.1±0.1	5.8±0.6
*tim-Gal4 Pdf-Gal80/UAS-ShawTR*	15	87	23.5±0.1	3.8±0.3
*tim-Gal4/UAS-ShawTR; cry-Gal80/+*	3	100	23.9±0.3	5.5±1.0
*tim-Gal4/UAS-ShawRNAi*	32	97	24.3±0.1	4.8±0.3
*tim-Gal4 Pdf-Gal80/UAS-ShawRNAi*	41	85	24.4±0.1	3.4±0.2
*Pdf-Gal4/UAS-ShawRNAi*	27	93	24.5±0.1	4.0±0.2
*tim-Gal4 ShawRNAi/+; cry-Gal80/+*	20	90	27.4±0.4	5.4±0.4
*tim-Gal4/+*	19	89	24.6±0.2	4.9±0.6
*cry-Gal4/+*	20	90	24.0±0.1	6.7±0.5
*Pdf-Gal4/+*	29	90	24.5±0.1	4.5±0.3
*cry-Gal4, Pdf-Gal80/+*	13	100	24.1±0.1	6.1±0.5
*tim-Gal4, Pdf-Gal80/+*	14	100	24.7±0.1	3.8±0.1
*UAS-Shaw/+*	23	100	23.7±0.1	5.9±0.2
*UAS-ShawTR/+*	15	100	23.5±0.1	4.5±0.3
*UAS-ShawRNAi/+*	29	93	23.6±0.1	6.5±0.4
*UAS-Shaw/+; cry-Gal80/+*	13	85	23.7±0.2	4.7±0.5

Individual male flies were first kept in light∶dark (LD) cycles for 7 days, before being released into constant darkness (DD). Free-running period length (τ) and the significance of the rhythms (RS) were determined by autocorrelation as described by Levine et al [33]. Flies with an RS value ≥2 were considered rhythmic. Errors indicate SEMs.

Arrhythmicity in *tim-GAL4/UAS-Shaw* flies is similar to that seen with over-expression of an open-rectifier K^+^ channel (dORK) in clock neurons [15]–[16]. However for dORK-induced arrhythmicity, expression in the PDF neurons is sufficient, whereas *Pdf-GAL4/UAS-Shaw* flies remain rhythmic (Figure 3B). Some flies show signs of possible internal desynchronization (e.g., upper panel in Figure 3B) but the effects are much milder than those resulting from LNv expression of other channel transgenes [17], [42].

*cry-GAL4/UAS-Shaw* flies, which express in fewer clock neurons compared to *tim-GAL4* (LNv, LNd, 4 DN_1_ and 2 DN3's) maintain rhythmic behavior (Figure 3C). This suggests that a spatially more restricted over-expression of Shaw does not grossly impair clock neuronal communication (Table 2 and Figure 3C). Removing Shaw over-expression in the LNvs only (*tim-GAL4, Pdf-GAL80/UAS-Shaw*) appears to result in a small increase of rhythmically behaving flies compared to expressing *Shaw* in all clock neurons (*tim-GAL4/UAS-Shaw*) suggesting that Shaw in the LNv influences DD behavior to some extent (Table 2). But given the drastic difference between *tim-GAL4* and *cry-GAL4* drivers, the *cry-GAL4* negative DN_1_, DN_2_ and DN_3_ seem to be the main cause for the observed behavioral arrhythmicity. To test if this is the case, we restricted *Shaw* expression to these cells (*tim-GAL4/UAS-Shaw; cry-GAL80*). Although not all the flies were arrhythmic in DD, more than 30% were, and the rhythmic flies exhibited rather weak rhythmicity (Figure 3E, Table 2). Therefore, as is the case for LD behavior, the DN neurons seem to be particularly sensitive to *Shaw* levels, and more importantly, they seem to be able to block rhythmic locomotor activity, even if the Lateral Neurons are not manipulated. Manipulations expected to increase electrical activity of all clock neurons (*tim-GAL4/UAS-ShawTR*; Table 2) also lead to reduced rhythmicity, but expression of *ShawRNAi* in all clock neurons caused no gross effect on rhythmicity in DD with any of the drivers (Table 2). However, reduction of *Shaw* expression in the DNs alone (*tim-GAL4 ShawRNAi/+; cry-GAL80/+*) lead to a dramatic period lengthening of 3.5 to 4 hr (Figure 3F, Table 2). This clearly shows that endogenous *Shaw* contributes to clock function in DD.

Interestingly, reducing *Shaw* function by means of the dominant negative *ShawTR* transgene did not result in a period lengthening when its expression was restricted to the DN cells (Table 2). Although only a few flies were tested, they exhibited robust and normal-period rhythms. This indicates a qualitative difference between reducing *Shaw* levels (RNAi) and introducing a dominant negative form of *Shaw*, which seems to multimerize with wild-type Shaw subunits in cytoplasmic organelles (Figure 1, [25]). Expression of Shaw-RNAi or Shaw-TR in clock neurons did not lead to any early mortality.

### Over-expression of *Shaw* does not interfere with basic molecular oscillations in clock neurons

**Figure 4:**
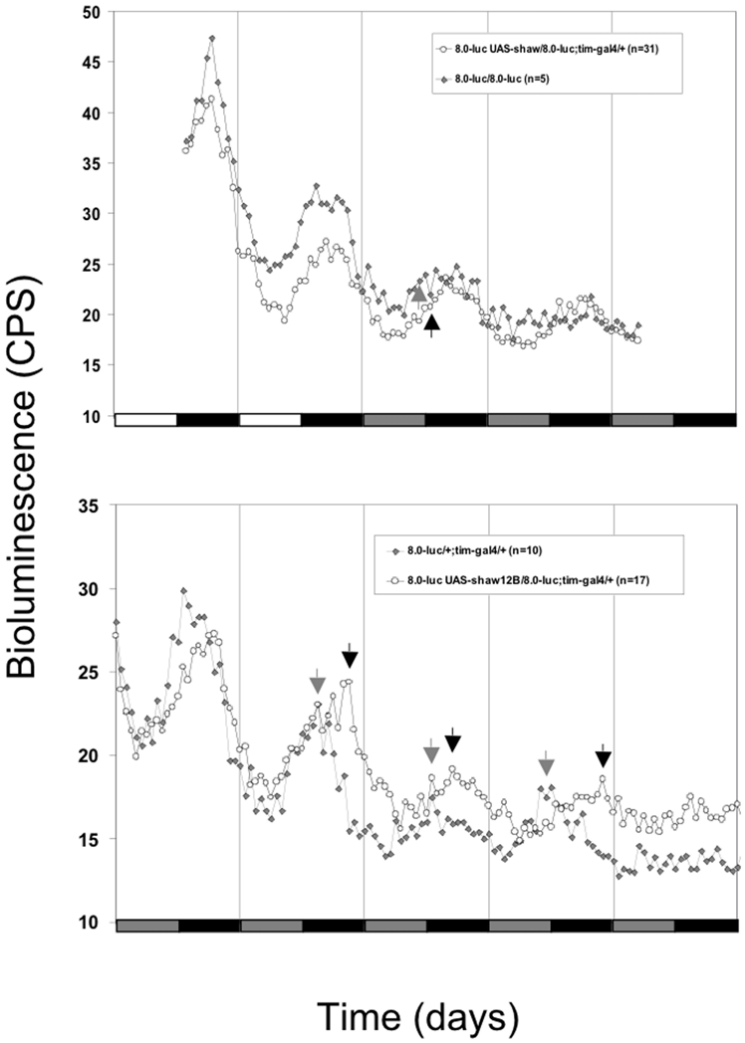
Over-expression of *Shaw* does not disrupt the molecular clock in Dorsal clock Neurons (DNs). Flies of the indicated genotypes were individually placed in micro titer plates containing food and luciferin and bioluminescence levels were measured automatically (Materials and Methods). Flies were initially exposed to two LD cycles followed by 3 (upper panel) and 5 (lower panel) days in constant darkness (DD). Average blots are shown. The *8.0-luc:9D* transgene (a promoter-less, Period-Luciferase encoding fusion gene) is predominantly expressed in the LNd, DN_1_, and DN_3_ neurons [30]. Note that expression of *Shaw* is correlated with a small increase in period length in both experiments. Black and grey arrows indicate the rising phase (upper panel), or peak (lower panel) of *luciferase* expression for the *Shaw* expressing and control flies, respectively. White bars indicate times when the lights were “on”, black bars indicate “lights off”. Grey bars signify times in constant darkness when the lights would have been “on” in a continued light∶dark cycle (subjective day).

In order to see if Shaw-mediated changes in clock electrical activity affect the molecular circadian rhythm we employed a luciferase reporter assay, which allows clock gene expression to be monitored in real time in live flies (e.g. [30], [32]). Because Shaw-mediated changes in the DNs seem to have the greatest effect on maintenance of rhythmic behavior in free running conditions (Table 1) we used a promoter-less, Period-Luciferase (Per-Luc) encoding fusion gene (the *8.0-luc:9D* transgene), in which (Per-Luc) is predominantly expressed in the DN_1_, DN_2_, and DN_3_ neurons with occasional expression in a small subset of the LNd neurons [30]. Flies were exposed to two LD cycles followed by either 3 or 5 days in constant darkness (Figure 4). Activation of *Shaw* expression in all *tim* expressing cells did not have any drastic effect on *8.0-luc* reported PER expression in the DNs, neither in LD nor in DD. This indicates that although all clock neurons are electrically inactivated, the molecular feedback loops operate more or less normally at least in the DNs. We did observe a slight increase in period length when *Shaw* was over-expressed in the *8.0-luc* background indicating some influence on the central clock mechanism (Figure 4, black arrows compared to control grey arrows), perhaps caused by a missing feedback to central clock neurons in behaviorally arrhythmic flies.

**Figure 5:**
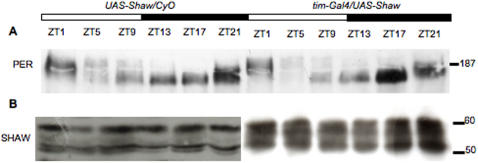
Peripheral clocks are not affected by over-expression of *Shaw.* Total head extracts were prepared from flies maintained under LD conditions and collected at the time (ZT, hours) indicated above each lane. Open or solid bars between blots indicate lights being on or off (e.g. ZT0 is the time of “lights on” and ZT12 is time of “lights off”). (A) To determine the time-dependent changes in mobility and abundance of PER in wild-type heads, equal numbers of *UAS-Shaw/CyO* heads (25) were loaded per lane and analyzed by Western blotting using anti-PER antibodies [32]. In order to determine the effect of over-expression of Shaw, equal numbers of *tim-GAL4/UAS-Shaw* heads (25) were loaded per lane and a similar change in temporal abundance and phosphorylation of PER was seen as in wild-type. (B) Robust Shaw over-expression is visible by comparing *tim-GAL4/UAS-Shaw* to *UAS-Shaw/CyO* on a separate nitrocellulose membrane incubated with anti-Shaw antibodies [25]. Due to the high quantity of Shaw in the over-expressing flies a shorter exposure of the *tim-GAL4/UAS-Shaw* half of the blot is shown and only 7 heads of this genotype were loaded (compared to 25 heads of the control). Three experiments were performed under these conditions with similar results.

It is possible that broader effects on the molecular clock in peripheral clock cells went unnoticed in our spatially restricted *luciferase* assay. We therefore asked if Shaw-mediated electrical silencing would affect molecular clock properties in the whole fly heads (largely reflecting clock gene expression in the compound-eye photoreceptor cells) and determined the abundance and phosphorylation state of endogenous PER (e.g., [32]). To do this we performed Western blots on proteins extracted from *tim-GAL4/UAS-Shaw* and control heads collected at different times during a LD cycle (Figure 5). Although we saw a robust over-expression of Shaw protein in *tim-GAL4/UAS-Shaw* heads compared to controls (Figure 5B), the relative abundance and phosphorylation state of PER during the LD cycle did not seem to differ between *tim-GAL4/UAS-Shaw* and control heads (Figure 5A). This result correlates well with the non-disturbance of *per-luciferase* rhythms by *Shaw* over-expression (Figure 4). Although we cannot rule out that the molecular clock is affected in LNv and LNd pacemaker neurons, we favor the idea that *Shaw* mis-expression specifically effects neuronal clock output (see below and Discussion).

### Shaw disrupts rhythmic accumulation and levels of PDF in neuronal pacemaker terminals

**Figure 6:**
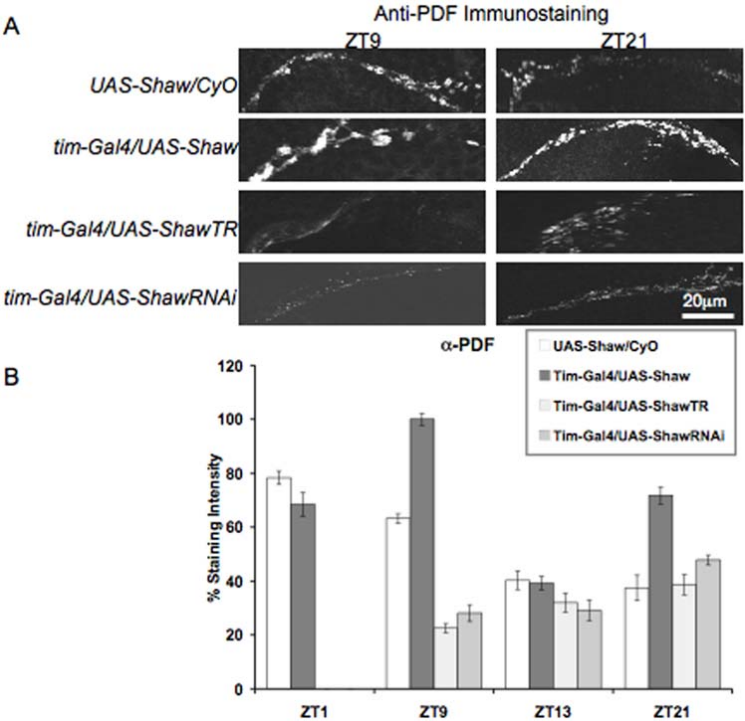
Changing Shaw levels in all clock neurons effects cyclic accumulation of PDF in LNv terminals. (A) Confocal image of the dorsal projections of the LNv neurons revealed by anti-PDF antibodies. The panels show representative samples of adult brains containing clock neurons with normal levels of Shaw (*UAS-Shaw/CyO*), elevated levels of Shaw (*tim-GAL4/UAS-Shaw*), a dominant-negative form of Shaw (*tim-GAL4/UAS-ShawTR*), or reduced levels of Shaw (*tim-GAL4/UAS-Shaw-RNAi*) that were fixed at the two time points indicated (ZT9 and ZT21). (B) Quantification of average staining intensity of the dorsal LNv projections from a single experiment (n = 7 to 10 hemispheres). Error bars represent SEM. In control brains PDF accumulation is higher during the day compared to the night. This circadian difference in PDF intensity is not seen when Shaw levels are manipulated in the clock neurons using *tim-GAL4*. In addition PDF tends to accumulate to higher levels compared to wild-type when Shaw levels are increased, while reduction or dominant-negative forms of Shaw tend to have the opposite effect.

A mechanism by which Shaw K^+^ channels could influence the output of the clock independent of any effect on the molecular rhythm is by affecting PDF release from clock neurons. PDF release from *Drosophila* clock neurons is circadianly regulated and controls output behavior [26]. In order to determine if changing the level of Shaw in clock neurons affects rhythmic accumulation of PDF in the dorsal projections of s-LNv neurons, we quantified PDF levels in LNv terminals in the dorsal brain during a LD cycle (Figure 6). In wild-type animals there is more PDF in the LNv terminals during the day (ZT1 or ZT9) than at night (ZT13 or ZT21; p<0.00001) (Figure 6A–B; cf [26]). This circadian variation in PDF intensity is abolished when Shaw levels are manipulated in the clock neurons using *tim-GAL4*. Shaw over-expression in all clock neurons results in overall increased PDF levels compared to wild-type (ZT1 p<0.05, ZT9 and ZT21 p<0.00001), which are approximately the same at ZT1 and ZT21, and reaching peak and trough levels at ZT9 or ZT13, respectively (p<0.00001). Expression of dominant negative Shaw causes lower PDF levels at day and night compared to wild-type (ZT9 p<0.00001), but levels are somewhat higher at ZT21 and ZT13 compared to ZT9 (p<0.001 and p<0.05 respectively). Reducing Shaw in all clock neurons using Shaw RNAi also results in an overall reduction of PDF levels (ZT9 p<0.00001, ZT13 p<0.05) and peak levels are also reached during the night (p<0.0001). We cannot rule out that there might still be some PDF rhythm after *Shaw* over-expression, and possibly still residual, but improperly phased rhythms in flies with reduced Shaw.

The results suggests that increasing hyperpolarizing Shaw throughout the clock circuit causes LNv to display decreased probability of neuropeptide release resulting in the accumulation of PDF in the LNv terminals. In turn, this could explain the increased LD activity levels displayed in this genotype. Genotypes with a reduction of functional Shaw throughout the clock have lower PDF levels in their LNv terminals, perhaps caused by an accelerated neuropeptide release, which in this case is not correlated with a reduction of behavioral activity.

**Figure 7:**
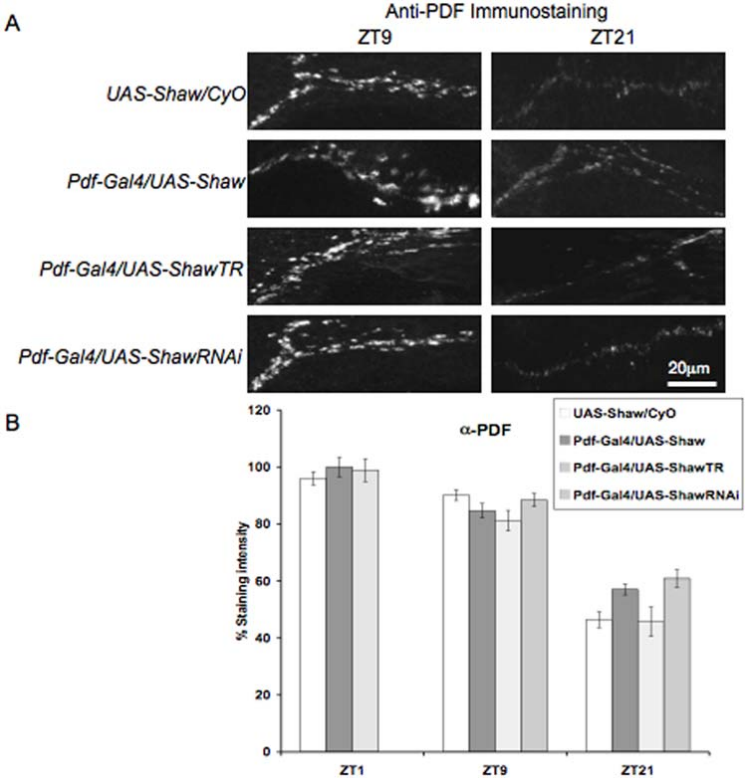
Changing Shaw levels in the LNv's only, does not effect cyclic accumulation of PDF. (A) Maximum projections are shown for confocal sections containing the dorsal projections of the LNv neurons revealed by anti-PDH antibodies. The panels show representative samples of adult brains containing clock neurons with normal levels of Shaw in the LNv (*UAS-Shaw/CyO*), elevated levels of Shaw (*Pdf-GAL4/UAS-Shaw*), a dominant-negative form of Shaw (*Pdf-GAL4/UAS-Shaw-TR*), or reduced levels of Shaw (*Pdf-GAL4/UAS-Shaw-RNAi*) that were fixed at two time points indicated (ZT9 and ZT21). (B) Quantification of average intensity of the LNv dorsal projections from a single experiment (n = 7 to 10 hemispheres). Error bars represent SEM. Brains from all genotypes display similar PDF levels at all times.

Surprisingly, when Shaw is manipulated only in the PDF expressing LNv, rhythmic accumulation of PDF is not disrupted. PDF in the LNv terminals remains higher during the day (ZT1 or ZT9) than at night (ZT21; p<0.00001), as in the controls (Figure 7A–B). Furthermore, the bidirectional change in absolute levels of PDF in LNv terminals is no longer observed. There is no change in absolute levels of PDF at ZT1 and ZT9 between genotypes. At ZT21 PDF levels in LNv terminals of *Pdf-GAL4/UAS-ShawTR* and wild-type are the same, while *Pdf-GAL4/UAS-Shaw* (p<0.01) and *Pdf-GAL4/UAS-ShawRNAi* (p<0.001) show weakly increased PDF abundance.

This suggests that changing Shaw levels in LNv neurons alone has little effect on either rhythmic accumulation or levels of PDF in the LNv terminals. These data are consistent with the lacking effect of changing Shaw levels in LNv neurons on locomotor activity under LD conditions. Instead it seems that changing Shaw levels in the DN cells has the greatest effect on locomotor activity (Figures 2–3; Tables 1–2) and rhythmic accumulation or levels of PDF levels in the LNv terminals (Figures 5–6). This implies that the DN may affect PDF release indirectly, pointing to a potential circuit level effect of Shaw mediated changes in electrical activity of the DN. For instance, the Shaw mediated change in electrical activity may cause release of an anterograde or retrograde signal that influences the LNv's rhythmic accumulation or level of PDF. Furthermore, the larger effect of changing Shaw levels in DN cells on locomotor activity maybe related to expression of the PDF receptor in these cells, suggesting that they have a role in receiving and transferring circadian signals to downstream neurons controlling locomotor activity [43]–[44].

## Discussion

We show that Shaw-mediated changes in clock neuronal electrical activity severely affect the fly's locomotor activity in LD and DD conditions. Mammalian Shaw homologues have been shown to have a central function in clock neurons, and were recently demonstrated to be the first circadianly-regulated intrinsic voltage-gated currents in mammalian cells [21].

In LD conditions, over-expression of *Shaw* K^+^ channels (electrical inactivation) results in an increase in locomotor activity at night. Therefore our data suggest that under LD conditions appropriate Shaw-mediated electrical activity is not required for rhythmic output from the clock, but rather regulates overall behavioral activity levels with the DN neurons being particularly important for this regulation. Similarly in SCN neurons of the mammalian system, decreased electrical activity is correlated with increased behavioral activity [5], [12].

In DD, over-expression of Shaw in clock neurons lead to arrhythmic behavior, consistent with previous reports, in which clock neurons were electrically silenced [15]–[16]. A dominant negative form of Shaw (ShawTR) lead to mild arrhythmia, but reduction by RNAi had no effect. This suggests that the underlying causes of Shaw RNAi-induced hyperexcitability are different for Shaw dominant-negative-induced hyperexcitability and may involve differences in localization of the channel or differences in compensatory changes in other channels that arise from over-expression of the two different transgenes.

Again as for Shaw mediated changes in LD, the Dorsal Neurons seem to have a special role in regulation of DD behavior and are particularly sensitive to changes in the level of Shaw. Restricting over-expression of Shaw to subsets of the Dorsal Neurons with *tim-GAL4/UAS-Shaw; cry-GAL80* (*cry-GAL80* has been shown to be effective in suppressing *tim-GAL4* driven expression in all LNs, but allowing expression in the majority of DNs [18], [28]–[29] caused flies to be arrhythmic in DD or to display weaker rhythms. Furthermore, reduction of *Shaw* expression in the DNs alone (*tim-GAL4 ShawRNAi/+; cry-GAL80/+*) caused a dramatic period lengthening of 3.5–4 hr, confirming that endogenous *Shaw* contributes to clock function under constant conditions.

The fact that we observe this phenotype only when manipulating *Shaw* in a subset of the clock neurons even though we find endogenous Shaw in all clock-neuronal groups, strongly suggests that *Shaw* may mediate neuronal properties of all clock neurons. However reducing *Shaw* levels equally throughout the whole clock neuronal network has little effect, perhaps because the communication between the individual cells with respect to each other is not changed and their electrical properties are all changed in the same direction. If only a subset of the network is altered (i.e., the DN), communication can be influenced or disrupted, resulting in long period rhythms. Alternatively, the clock may be able to compensate for equal increases in electrical activity throughout its neural circuit and preserve its functional output. If however activity is only changed in part of the hierarchy of clock neurons (the DNs), then the clock circuit cannot compensate as a whole and behavior becomes compromised, in this case indicated by a lengthening of the free-running period.

Our results are similar to those obtained after blocking pacemaker synaptic output with tetanus toxin (ttx) [45]. First, in DD, expression of either ttx or Shaw does not cause behavioral arrhythmicity when expressed in PDF neurons alone. Both must be expressed in all clock neurons or in all PDF*^−^* clock neurons to cause arrhythmicity. Second, blocking output of TIM but not PDF neurons by either transgene increases activity at night during LD conditions. This correlation suggests that Shaw somehow affects chemical signaling between clock neurons in non-PDF cells. In turn this could explain the alteration of PDF accumulation in LNv terminals observed with *tim-GAL4* (but not *Pdf-GAL4*) driven Shaw over-expression and down-regulation.

Shaw expression in all *tim* expressing cells had no gross effects on PER oscillations in LD in peripheral clock cells (Figure 5), nor did it interfere with self-sustained rhythmic PER expression in the DNs (Figure 4). This suggests that endogenous Shaw is important for regulated output from the clock neurons but not for sustaining clock molecular feedback loops. Perhaps Shaw controls RMP in clock neurons in a circadian fashion and hence affects the rhythmic release of neuropeptides such as PDF or other neurotransmitters from clock neurons. Indeed we find that altering Shaw levels in clock neurons disrupts the cyclic accumulation of PDF in the dorsal projections of the LNv neurons. Increasing Shaw in all clock neurons causes PDF accumulation in small LNv terminals possibly by causing a decrease in the probability of neuropeptide release. This suggests that Shaw-dependent changes in the LNd and DNs are responsible for rhythmic accumulation of PDF, which in turn may mediate changes in LD locomotor activity. This result also suggests that Shaw regulation of PDF release is not cell-autonomous and may result from an indirect effect of the LNd and DN on the LNv terminals. Alternatively, the relevant LNd and DN cells express the PDF receptor and are therefore important for the transfer of the PDF signal to downstream neurons controlling locomotor activity.

It is not understood how temporal information from the molecular clock in the nucleus is conveyed to the membrane, and how rhythmic physiological events in the membrane feedback to the molecular clock. So far this question has been addressed by expression of *dORK* in LNv neurons [15]–[16], which results in behavioral arrhythmicity in DD and loss of molecular clock protein cycling. This is surprising because in cultured mammalian SCN clock neurons, reversal of action potential blockade by tetrodotoxin allows circadian firing rhythms to return with the exact phase as when the tetrodotoxin was added [46]. This suggests that in the SCN, the clock still runs even when it is decoupled from membrane properties. Similarly in flies, blockade of synaptic release in all clock neurons (*tim*-GAL4/UAS-*ttx*) caused DD arrhythmia but TIM cycling in all clock neuronal types (except for the l-LNv, who stop cycling immediately after transfer to DD) remained normal in *tim*-GAL4/UAS-*ttx* flies under LD and DD [45].

Another study that addressed the relationship between electrical activity and the molecular clock of *Drosophila* used a series of mutations in *narrow abdomen* (*na*), another channel encoding gene expressed in clock neurons [18]. *na* mutations would be expected to hyperpolarize RMP and result in DD behavioral arrhythmicity. However unlike the case of *dORK*, this was not correlated with a loss of molecular clock protein cycling [18]. It is not clear why Nitabach et al. [15] saw a disruption of PER oscillations in flies with electrically silenced clock neurons, but we and others have not. It is possible that expression of dORK channels has a more profound effect on neuronal membrane physiology than any other manipulation. But this seems unlikely as both panneural expression of Shaw [25] and dORK [15] result in lethality, while pan-clock-neuronal expression of both channels results in arrhythmicity (Figure 3 this study; and Figure 6 [16]). There are experimental differences between their study and ours that may explain the contradictory results. Nitabach et al. [15] electrically inactivated only the LNv neurons with an exogenous Open Rectifier K^+^ channel (dORK) and then used semi-quantification of intensity of PER and TIM antibody staining of the LNvs to judge the status of the molecular clock. In contrast, we electrically inactivated all clock neurons using an endogenous non-inactivating K^+^ channel and directly measured *per-luciferase* oscillations of the DNs in the intact animal.

Based on these studies, it seems uncertain if changes of membrane properties feedback to influence circadian clock gene expression and if they are indeed required for molecular clock function. If true, this would imply that in *Drosophila* membrane properties may only influence clock output. In addition, the fact that we observed Shaw-dependent period-lengthening of behavioral rhythms and PER oscillations suggests that altered membrane properties may *modulate* the molecular clock. Such a Shaw-dependent lengthening in circadian period in our luciferase or locomotor activity data also reiterates a key current concept in the study of circadian systems: the temporal readout to behavior does not dependent solely on a cell autonomous molecular timekeeper, but coordinated clock output is a property that arises from many clock cells acting together in concert with electrical activity and neurochemicals mediating intercellular signaling and synchronization. In this respect it will be interesting to determine the molecular period of the different subsets of clock neurons in the long period flies caused by reduced *Shaw* levels in the DN only.

Our data lends further support to recent studies that show the electrical activity of *Drosophila* clock cells is circadianly regulated with resting membrane potential and associated electrophysiological parameters varying between subjective day and subjective night [47]. It has also been shown that clock cell electrical activity affects the coordination of molecular oscillations between clock cells by both PDF-dependent and independent factors [17]. When compared to our current understanding of the SCN network [48] these findings along with another recent *Drosophila* study that demonstrated that intracellular Ca^2+^ regulates clock cell oscillations [49], would suggest that many physiological in addition to molecular mechanisms of circadian clocks are evolutionary conserved between the *Drosophila* brain clock and the mammalian SCN.

Under free-running conditions appropriate levels of Shaw-mediated electrical activity are required in the clock network to maintain rhythmic activity, whereby the DN (especially the *cry-GAL4* negative DN1-3) appear to be particularly important for this behavior. Alternatively (and especially in respect to the PDF staining results), the s-LNvs could be the “most important” Shaw cells, but the *cry-GAL4* and *Pdf-GAL4* drivers maybe too weak (compared to *tim-GAL4*) to observe an effect of Shaw expression on the circadian clock. Either way our data suggest that PDF negative clock neurons participate in regulation of DD behavior. The importance of the DN in circadian rhythms has been reiterated in recent studies demonstrating that these cells regulate rhythmic behavior in LL [29], [40] and are also important for relaying PDF receptor mediated signals to cells regulating circadian output behavior [43]–[44].

Based on our results it is likely that Shaw modulates electrical activity of (DN) clock neurons, which control circadian locomotor behavior by affecting rhythmic release of PDF. We have demonstrated a central role of Shaw K^+^ channels for coordinated and rhythmic output from clock neurons in *Drosophila*, which seems to be conserved in mammals, where homologues of Shaw have also been implicated in controlling circadian behavior [21]. Future studies will hopefully reveal which molecules connect the molecular clock with Shaw's neuronal membrane function.

## Supporting Information

Figure S1 Behavior of flies in light: dark cycles over-expressing Shaw or a dominant-negative form of Shaw in different subsets of clock neurons. Histograms show daily averages of locomotor activity in the LD portion of the experiment (7 days). Open and black bars indicate activity levels during 30 min intervals when the lights were on and off, respectively. All genotypes exhibit bimodal behavior, showing the characteristic anticipation of the lights-on transition in the morning, and the lights-off transition in the evening. Note that only the *cry-GAL4/Shaw* flies exhibit a Shaw-dependent activity increase during the night (see text and Table 1 for details). SEM's are indicated by dots above each histogram bar.
(6.01 MB TIF)
