# Supplementary figures and images for: Function of the Shaw Potassium Channel within the Drosophila Circadian Clock
Source: PLoS One. 2008 May 28;3(5):e2274. doi: 10.1371/journal.pone.0002274 (PMC2386553; doi:10.1371/journal.pone.0002274)

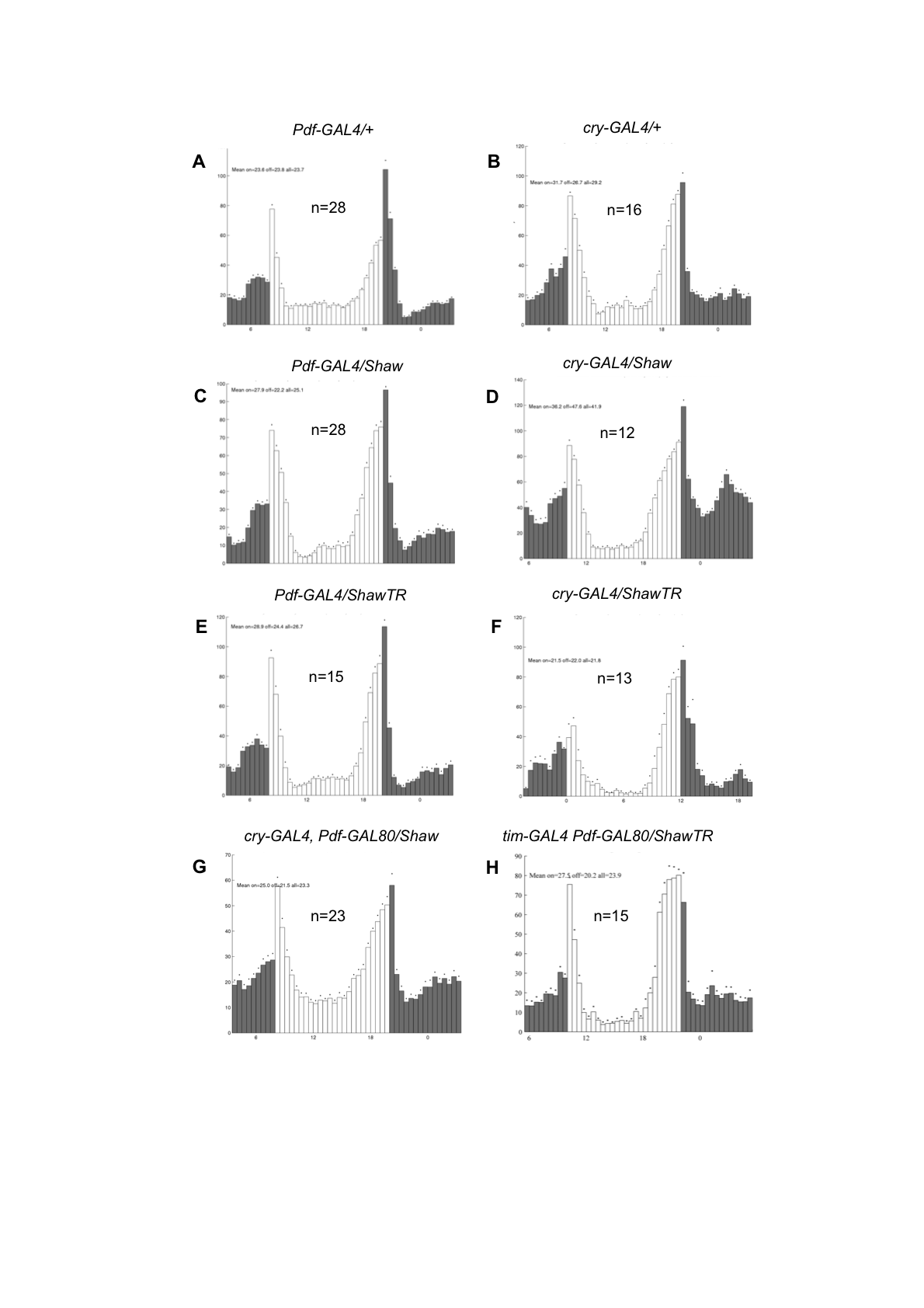

Supplement: Figure S1 — Behavior of flies in light: dark cycles over-expressing Shaw or a dominant-negative form of Shaw in different subsets of clock neurons. Histograms show daily averages of locomotor activity in the LD portion of the experiment (7 days). Open and black bars indicate activity levels during 30 min intervals when the lights were on and off, respectively. All genotypes exhibit bimodal behavior, showing the characteristic anticipation of the lights-on transition in the morning, and the lights-off transition in the evening. Note that only the cry-GAL4/Shaw flies exhibit a Shaw-dependent activity increase during the night (see text and Table 1 for details). SEM's are indicated by dots above each histogram bar. (6.01 MB TIF) [file pone.0002274.s001.tif]
